# Tyr76 is essential for the cold adaptation of a class II glutaredoxin 4 with a heat‐labile structure from the Arctic bacterium *Sphingomonas* sp.

**DOI:** 10.1002/2211-5463.13560

**Published:** 2023-01-29

**Authors:** Trang Hoang, ChanSu Jeong, Sei‐Heon Jang, ChangWoo Lee

**Affiliations:** ^1^ Department of Biomedical Science and Center for Bio‐Nanomaterials Daegu University Gyeongsan South Korea

**Keywords:** aromatic amino acids, cis‐proline loop, cold adaptation, glutaredoxin, hydrogen bond network, thioredoxin‐fold

## Abstract

Glutaredoxins (Grxs) are small proteins that share a well‐conserved thioredoxin (Trx)‐fold and participate in many biological processes. This study examined the cold adaptation mechanism of a Fe‐S cluster binding class II Grx4 (SpGrx4) from the psychrophilic Arctic bacterium *Sphingomonas* sp. PAMC 26621. Three polar residues close to the cis‐proline residue (P73) of SpGrx4 form a hydrogen bond network (Q74–S67–Y76) with the cis‐proline loop main chain. The hydroxyl group of S67 or Y76 or both is replaced in similar Grxs depending on the temperature of the habitat. Mutants with reduced hydrogen bonds (S67A, Q74A, Y76F, and S67A/Y76W) were more susceptible to urea‐induced unfolding and more flexible than the wild‐type (WT). By contrast, Y76W, with a bulky indole group, was the most stable. These mutants showed higher melting temperatures than WT as a consequence of increased hydrophobic interactions. These results suggest that the tyrosine residue, Y76, is preferred for the cold adaptation of SpGrx4 with a heat‐labile structure despite the rigid cis‐proline loop, due to hydrogen bond formation. An aromatic residue on β3 (cis‐proline plus3) modulates the stability‐flexibility of the cis‐proline loop for temperature adaptation of prokaryotic class II Grx4 members via hydrogen bonds and hydrophobic interactions.

## Graphical abstract

Members of the Fe‐S cluster binding class II glutaredoxin 4 family possess different aromatic amino acids on the β3‐strand (cis‐proline plus3) depending on the temperature of the habitat to stabilize the cis‐proline loop. A tyrosine residue with a phenyl hydroxyl group is preferred at lower and higher temperatures, as it compensates for the reduced hydrophobicity via hydrogen bond formation.

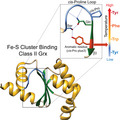

**Abbreviations**
G6PD: glucose 6‐phosphate dehydrogenase
Grx: glutaredoxin
GST: glutathione *S*‐transferase
Trx: thioredoxin

Cold‐adapted enzymes exhibit high activity at colder temperatures, but they are vulnerable to denaturation at warmer temperatures resulting from inherently flexible structures [1, 2, 3]. Cold‐adpated enzymes have undergone structural changes to maintain their flexible structure, including reduced intramolecular noncovalent bonds, extension of loop length, and increased access to active sites compared with their mesophilic counterparts [1, 4].

Grxs are ubiquitous small proteins belonging to the Trx‐fold superfamily that interact with glutathione [5, 6, 7, 8]. Class I Grxs (CxxC/S motif) serve as oxidoreductases, while class II Grxs (CGFS motif) bind Fe‐S clusters and show negligible oxidoreductase activity [6, 9]. The psychrophilic Arctic bacterium *Sphingomonas* sp. PAMC 26621 isolated from Svalbard Islands has two Grx subfamilies: the class I dithiol Grx3 with 85 amino acids (SpGrx3) and the class II monothiol Grx4 with 110 amino acids (SpGrx4) [10]. SpGrx3 and SpGrx4 share a canonical Trx‐fold consisting of the four‐stranded β‐sheet flanked by three α‐helices, but SpGrx4 has an additional α‐helix at the N‐terminus (Fig. 1A). The central β‐sheet of Grxs, which forms the hydrophobic core of the protein with the α‐helices, separates the hydrophobic core into two clusters on either side of the β‐sheet, as shown in Trxs [11, 12, 13]: one comprising aliphatic residues (aliphatic cluster) and the other comprising aromatic residues (aromatic cluster) (Fig. 1A and Fig. S1). The cis‐proline loop, which is approximately seven amino acids long and located near the active site of the Trx‐fold in the three‐dimensional space [14, 15, 16], participates in the interactions with glutathione via short antiparallel β‐sheet packing in Grxs [17]. The cis‐proline loops of prokaryotic class I and II Grxs are stabilized by a hydrogen bond network, involving the cis‐proline loop mainchain, a glutamine residue (cis‐proline plus1), and an amino acid containing an aromatic ring located in the aromatic cluster, such as a histidine residue (H63) on β4 in SpGrx3 (Fig. 1B and Fig. S2) or a tyrosine residue (Y76, cis‐proline plus3) on β3 in SpGrx4 (Fig. 1A). Multiple sequence alignment showed that the class II Grx4 members possess different aromatic residues on β3 (cis‐proline plus3) depending on the temperature of the habitat: a tyrosine residue in extremophiles (psychrophiles and thermophiles), a tryptophan residue in psychrotrophs and mesophiles, and a phenylalanine residue in moderate thermophiles (Fig. 1C). These variations in the aromatic residue on β3 are of particular interest in studying the temperature adaptation mechanism of prokaryotic class II Grx members. The hydrophobic force is temperature dependent, with its highest point between 30 °C and 80 °C, and becomes weaker at lower and higher temperatures [32].

**Fig. 1:**
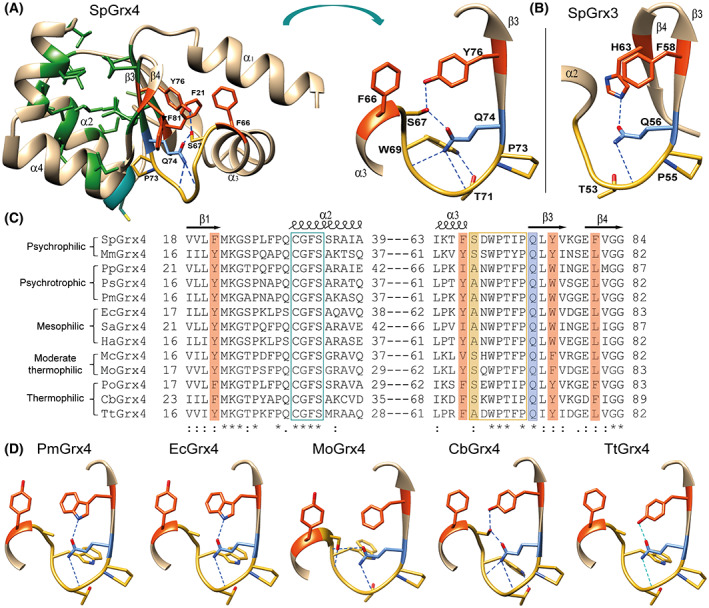
Cis‐proline loop structure of the class I SpGrx3 and class II Grx4 members and multiple sequence alignment. (A) The structural model of SpGrx4 with aliphatic residues in the aliphatic cluster (shown in green) and aromatic residues in the aromatic cluster (shown in orange) (left). Enlarged view of the cis‐proline loop structure of SpGrx4 (right). (B) Enlarged view of the cis‐proline loop structure of SpGrx3. (C) Multiple sequence alignment of Grx4 members with different temperatures of the habitat. (D) Comparison of the cis‐proline loop structure in Grx4 members. Structural models were built using alphafold except EcGrx4 drawn from 2WCI.pdb. The sequences are SpGrx4 (*Sphingomonas* sp. PAMC 26621, WP_010164075.1), MmGrx4 (*Marinobacter maritimus*, WP_144779181.1), PpGrx4 (*Psychrobacter phenylpyruvicus*, WP_028858520.1), PsGrx4 (*Pseudomonas stutzeri*, WP_103457808.1), PmGrx4 (*Pseudomonas mandelii*, WP_094470901.1), EcGrx4 (*Escherichia coli*, WP_001764546.1), SaGrx4 (*Staphylococcus aureus*, RTY95721.1), HaGrx4 (*Haemophilus*, WP_005653629.1), McGrx4 (*Methylococcus capsulatus*, WP_218800341.1), MoGrx4 (*Methyloterricola oryzae*, WP_200892121.1), PoGrx4 (*Porphyrobacter* sp., MBI1404354.1), CbGrx4 (*Chloroflexi bacterium*, MAT07599.1), and TtGrx4 (*Thermochromatium tepidum* ATCC 43061, QGU32454.1).

This study examined the contribution of the hydrogen bond network involving S67 and Q74 in the cis‐proline loop and Y76 on β3 (Q74–S67–Y76) with the cis‐proline loop mainchain to the cold adaptation of SpGrx4. Site‐directed mutagenesis was performed for SpGrx4 to generate a mutation of S67A (as shown in thermophilic TtGrx4), Q74A, Q74E, Y76F (as shown in moderate thermophilic MoGrx4), Y76W, and S67A/Y76W (as shown in psychrotrophic PmGrx4 and mesophilic EcGrx4) [19, 20, 21].

## Materials and methods

### Gene cloning and site‐directed mutagenesis

*Sphingomonas* sp. PAMC 26621 was kindly provided by the Polar and Alpine Microbial Collection of the Kore Polar Research Institute (Incheon, South Korea) [22]. The 333‐bp *grxD* (*spgrx4*) gene (NCBI ID: WP_010164075.1) was amplified from the genome of *Sphingomonas* sp. PAMC 26621 by PCR and subcloned into a TA vector (Enzynomics, Daejeon, South Korea). The TA–*spgrx4* construct, digested by *Nde* I and *BamH* I, was subcloned into a pET28 vector (Novagen, Madison, WI, USA) and transformed into *Escherichia coli* BL21 (DE3). The pET28–*spgrx4* construct was used as a template for site‐directed mutagenesis by PCR using *pfu* polymerase (PCR primers listed in Table S1). The PCR products were incubated with *Dpn* I at 37 °C for 1 h to remove the template plasmids before transformation into *E*. *coli* BL21 (DE3). The DNA sequence of the WT and mutant plasmids was confirmed by DNA sequencing.

### Bioinformatics

pymol (Schrodinger, LLC, New York, NY, USA) and ucsf chimera 1.16 were used to show the protein structures and interactions among the amino acids [23]. alphafold was used to build structural models of Grx members [24]. Multiple sequence alignment was performed using clustal omega [25].

### Expression and purification of SpGrx4 WT and mutant proteins

A single colony of *E*. *coli* BL21 (DE3) was grown overnight in a Luria–Bertani (LB) medium with 100 μg·mL^−1^ of kanamycin. The overnight culture was inoculated into 200 mL of freshly prepared LB medium (1 : 100 dilution) containing kanamycin. After reaching an optical density of 0.6–0.8 at 37 °C, isopropyl β‐d‐1‐thiogalaninectopyranoside (1 mm final concentration) was added to the LB medium. The culture was grown for an additional 20 h at 30 °C. The cells were harvested by centrifugation and washed with buffer A (50 mm Tris·HCl, 50 mm NaCl, 5 mm imidazole, and 0.1 mm ethylenediaminetetraacetate [EDTA]). The cell pellets containing SpGrx4 were resuspended in buffer A, followed by sonication on ice. After centrifugation at 10 000 × *g* for 30 min at 4 °C, the supernatant was collected and loaded into a 1‐mL HisTrap column (GE Healthcare, Piscataway, NJ, USA) equilibrated with buffer A. The nonspecific proteins were washed with buffer A containing 20 mm imidazole, followed by elution of the target proteins by buffer B containing 300 mm imidazole. All fractions containing SpGrx4 were loaded into a 1‐mL Capto Q column on an AKTA explorer system (GE Healthcare). The column was equilibrated with buffer C (50 mm Tris·HCl and 50 mm NaCl, pH 8.0). The recombinant Grx proteins were washed with 100–150 mm NaCl in buffer D (50 mm Tris·HCl and 1000 mm NaCl, pH 9.5) and eluted with a linear gradient of 170–500 mm NaCl in buffer D. All fractions containing the target proteins were desalted using the HiTrap desalting column and pooled in buffer E (20 mm Tris·HCl and 100 mm NaCl, pH 7.5). The purified SpGrx proteins were stored at −80 °C until needed.

### Denaturation curves

Urea‐induced protein unfolding was evaluated by measuring the fluorescence of tyrosine and tryptophan residues as a function of the denaturant concentration on a Scinco FS‐2 fluorescence spectrometer (Seoul, South Korea). The samples contained 0.8 μm protein and different urea concentrations (0–6.0 m) in buffer F (100 mm potassium phosphate, pH 7.4). The samples were incubated at 25 °C for 30 min before the measurements (excitation at 280 nm and emissions between 300 and 400 nm). Urea‐induced unfolding curves were analyzed, as described by Pace and Scholtz [26]. The fraction unfolded (*f*_U_) was calculated from the equation, *f*_U_ = (*y*_F_ − *y*)/(*y*_F_ − *y*_U_), where *y*_F_ and *y*_U_ represent the fluorescence values for the folded and unfolded states, respectively, under the conditions where the observed fluorescence value *y* is being measured [26].

### Quenching of protein fluorescence by acrylamide

The quenching of protein fluorescence was measured using a Scinco FS‐2 fluorescence spectrometer with increasing acrylamide concentration (0–0.4 m) in buffer E for 2 min at 25 °C (excitation at 280 nm and emissions between 300 and 400 nm). The acrylamide Stern–Volmer plots are presented as the ratio of intrinsic fluorescence intensity in the absence of acrylamide (*F*_0_) to fluorescence intensity in the presence of acrylamide (*F*).

### Protein thermal shift analysis

A thermal shift assay using SYPRO orange dye (Thermo Fisher Scientific, Waltham, MA, USA) was performed in 96‐well PCR plates on an Applied Biosystems StepOnePlus real‐time PCR instrument (Thermo Fisher Scientific) in the 25–99 °C range in 1 °C·min^−1^ increments. The proteins (0.6 mg·mL^−1^) were added to buffer E containing 100× SYPRO orange dye in a total volume of 20 μL. The melting temperature (*T*_m_) was determined using protein thermal shift software v1.4 (Thermo Fisher Scientific).

### Circular dichroism spectroscopy

The far‐UV circular dichroism (CD) spectra were measured using a JASCO J‐1500 spectropolarimeter at the Center for Scientific Instruments, Kyungpook National University (Daegu, South Korea). The samples at a final concentration of 0.3 mg·mL^−1^ in buffer E were incubated at 4 °C for 1 h, prior to the CD measurements. The α‐helical content of each protein was calculated using the K2D3 server [27].

## Results

### Expression and protein purification

The recombinant SpGrx4 proteins with the N‐terminal 6×His‐tag (WT, S67A, Q74A, Y76F, Y76W, and S67A/Y76W) were expressed as the soluble proteins in *E. coli* BL21 (DE3). They were purified to homogeneity using nickel‐chelate affinity chromatography and Capto Q anion‐exchange column chromatography (Fig. S3). On the contrary, Q74E failed to be expressed in *E. coli*. SpGrx4 WT and mutants appeared as a monomer in solution with a molecular weight of 14.2 kDa, as determined by size‐exclusion chromatography using Supderdex 200 column (data not shown).

### Effect of mutations on conformational stability

**Fig. 2:**
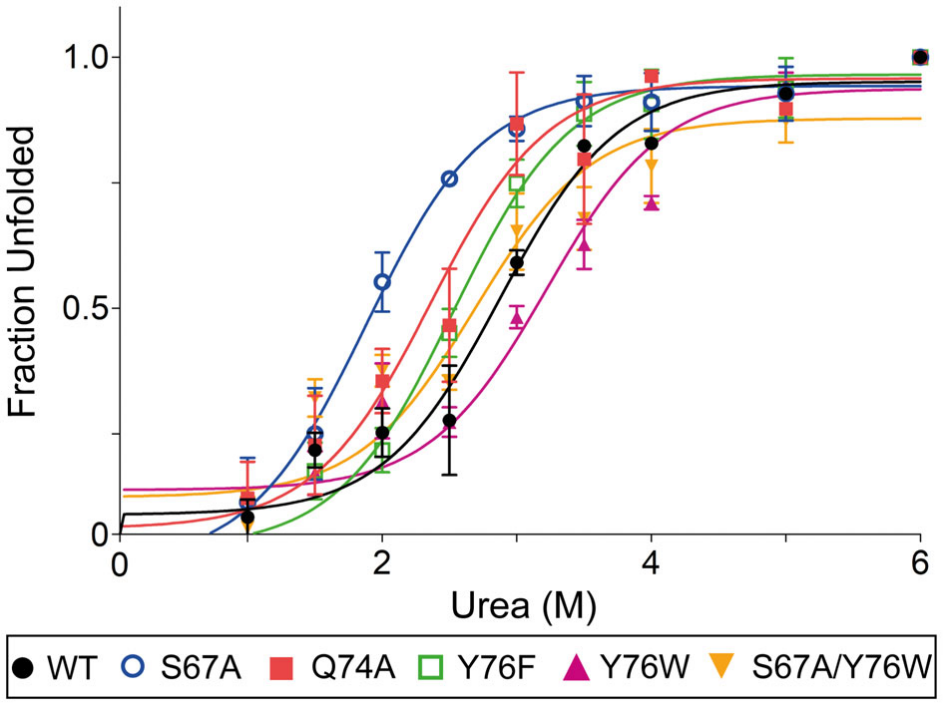
Urea‐induced unfolding of SpGrx4 WT and mutants. The chemical stability of SpGrx4 WT and mutants was assessed by measuring intrinsic protein fluorescence (excitation 280 nm) after incubation of the proteins at various urea concentrations (0–6 m) at 25 °C for 30 min. Data presented are means ± SD of three experiments. The values for the unfolded fraction were calculated using the equation in Materials and methods.

Urea‐induced protein unfolding was performed at 25 °C using fluorescence spectroscopy to evaluate the conformational stability of SpGrx4 WT and mutants (Fig. 2). An excitation wavelength of 280 nm was used because SpGrx4 has two tyrosine residues and one tryptophan residue (W69, Y76, and Y92). As urea disrupts the hydrogen bonds in the protein structure, the mutants with reduced hydrogen bonds (S67A, Q74A, Y76F, and S67A/Y76W) were more susceptible to denaturation with increasing urea concentration than WT. In particular, S67A and Q74A showed the lowest midpoints of urea denaturation (2.00 and 2.12 m, respectively), those of Y76F and the double mutant S67A/Y76W were lower (2.44 and 2.46 m, respectively) than WT (2.65 m). On the contrary, Y76W, with a bulky indole group, was the most stable (denaturation midpoint of 3.18 m) with hydrogen bonds between Q74–S67–W76. These results suggest that the hydrogen bond network in the cis‐proline loop determines the conformational stability of SpGrx4. The more stable structure of Y76W in urea unfolding compared to WT suggests that hydrophobic interactions of the side chain also contribute to the conformational stability of the protein.

### Effect of mutations on structural flexibility

**Fig. 3:**
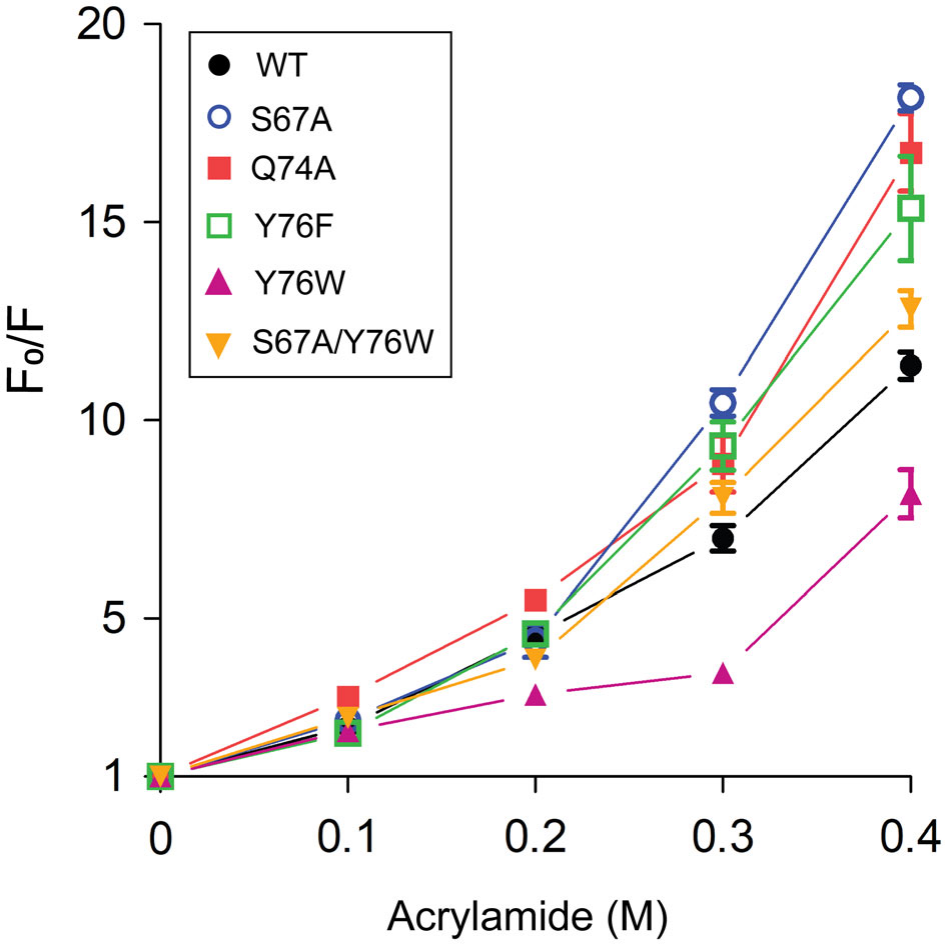
Structural flexibility of SpGrx4 WT and mutants. Acrylamide‐induced quenching of protein fluorescence was measured after incubation of the proteins at increasing acrylamide concentrations (0–0.4 m) for 2 min at 25 °C. The change in fluorescence intensity with acrylamide is shown as the ratio of the maximum fluorescence intensity without acrylamide (*F*_0_) to that under increasing acrylamide concentrations. The data presented are the means ± SD of three experiments.

Fluorescence quenching using acrylamide has been used to assess the flexibility of cold‐adapted enzymes because acrylamide penetrates psychrophilic enzymes more efficiently than its mesophilic and thermophilic counterparts [28]. The quenching of protein fluorescence by acrylamide was measured at 25 °C with increasing acrylamide concentration (excitation at 280 nm) (Fig. 3). The quenching of protein fluorescence (*F*_0_/*F*) was more apparent in S67A and Q74A with a small aliphatic alanine. Aromatic residue substitutions showed flexibility in the following order: Y76F > S67A/Y76W > WT > Y76W. Overall, disrupting the hydrogen bond network in the cis‐proline loop leads to a more flexible structure. The results suggest that the hydrogen bond network and hydrophobic interactions involving the aromatic residue on β3 (cis‐proline plus3) contribute to the structural flexibility of the protein. These results are consistent with those of urea‐induced protein unfolding (Fig. 2).

### Effect of mutations on thermal stability

The *T*_m_ value, at which 50% of the protein is unfolded, was determined for SpGrx4 WT and mutants using SYPRO orange‐based thermal shift analysis. The *T*_m_ values were Q74A (56.78 °C) > Y76W (50.17 °C) > Y76F (49.68 °C) > S67A (47.79 °C) > S67A/Y76W (46.06 °C) > WT (41.52 °C) (Table 1 and Fig. S4). Among the mutants, Q74A had the highest *T*_m_ value, which is 15.3 °C higher than WT, whereas the double mutant S67A/Y76W showed the lowest *T*_m_ value, 4.6 °C higher than WT. Despite the disruptions in the hydrogen bond network in the cis‐proline loop, these mutants showed an increase in melting temperatures by mutation‐induced hydrophobic interactions. The similar *T*_m_ values of Y76W and Y76F suggest that the more hydrophobic tryptophan and phenylalanine residues on β3 provide higher thermal stability than WT with a tyrosine residue.

**Table 1:** Midpoints of the urea denaturation curves and melting temperatures (*T*_m_) of SpGrx4 WT and mutants. The data presented are the means ± SD of three experiments.

	Midpoint of urea denaturation (m)	*T*_m_ (°C)
WT	2.65 ± 0.06	41.52 ± 0.14
S67A	2.00 ± 0.12	47.79 ± 0.15
Q74A	2.12 ± 0.28	56.79 ± 0.18
Y76F	2.44 ± 0.31	49.68 ± 0.04
Y76W	3.18 ± 0.10	50.17 ± 0.25
S67A/Y76W	2.46 ± 0.03	46.06 ± 0.17

**Fig. 4:**
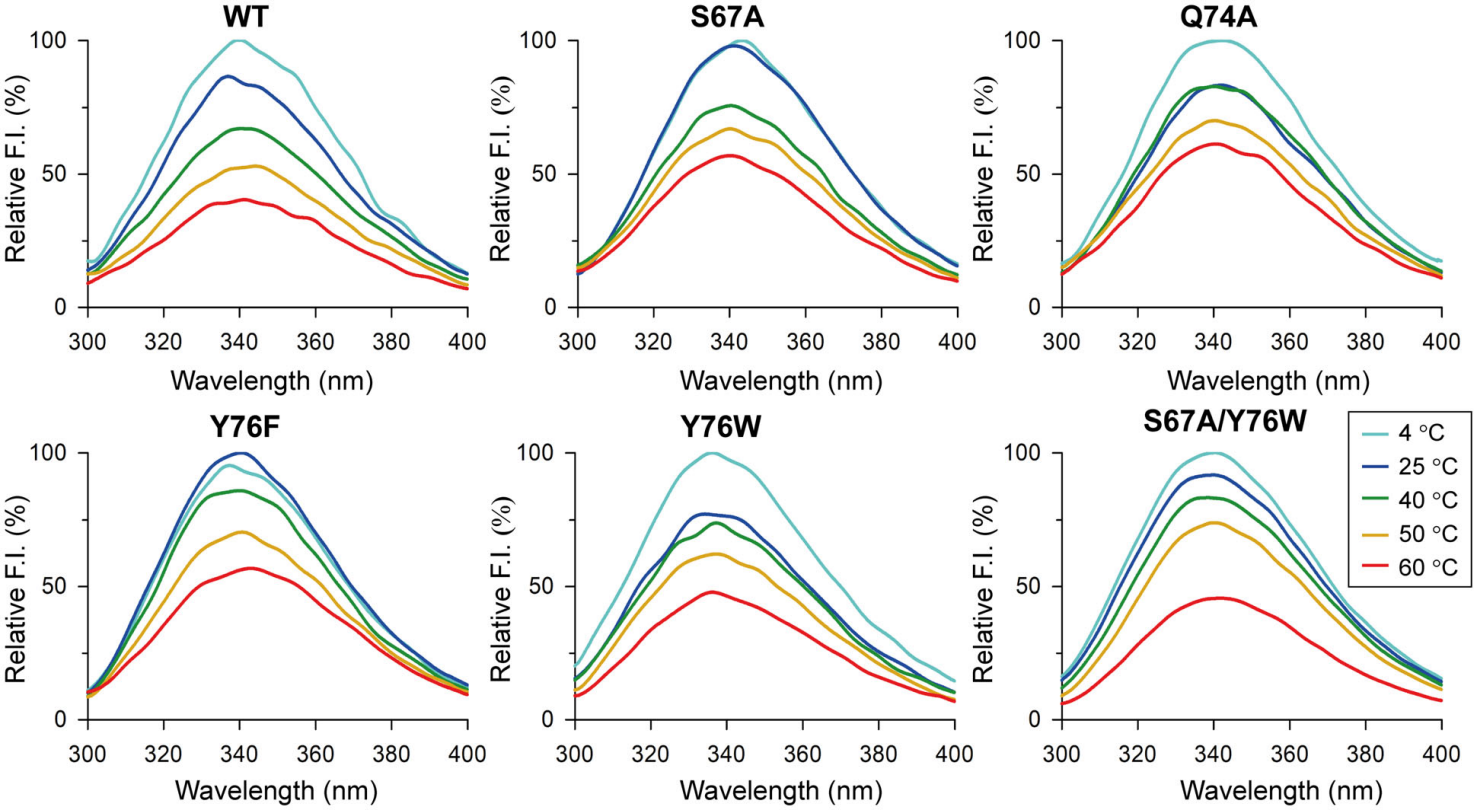
Thermal stability of SpGrx4 WT and mutants. The temperature‐induced unfolding of SpGrx4 proteins was evaluated by measuring intrinsic fluorescence after incubating the proteins at various temperatures (4–60 °C) for 1 h (excitation: 280 nm). The data presented are the means of three experiments.

The thermal stability of SpGrx4 WT and mutants was also evaluated by measuring intrinsic fluorescence intensity upon incubation of the proteins at 4–60 °C for 1 h (excitation at 280 nm) (Fig. 4). The fluorescence intensity ratios of WT decreased with increasing temperature. On the contrary, these mutants showed higher fluorescence intensity ratios than WT, resulting from hydrophobic substitutions in the cis‐proline loop. In particular, S67A and Y76F showed similar relative fluorescence intensity at 4 °C and 25 °C. By contrast, Q74A and Y76W showed a similar relative fluorescence intensity at 25 °C and 40 °C. The fluorescence intensity ratios of the double mutant S67A/Y76W were similar to those of WT, which differs from the single mutant S67A or Y76W. These results show that hydrophobic substitutions contribute to higher melting temperatures of SpGrx4 (Table 1).

### Changes in the secondary structure by mutations

**Fig. 5:**
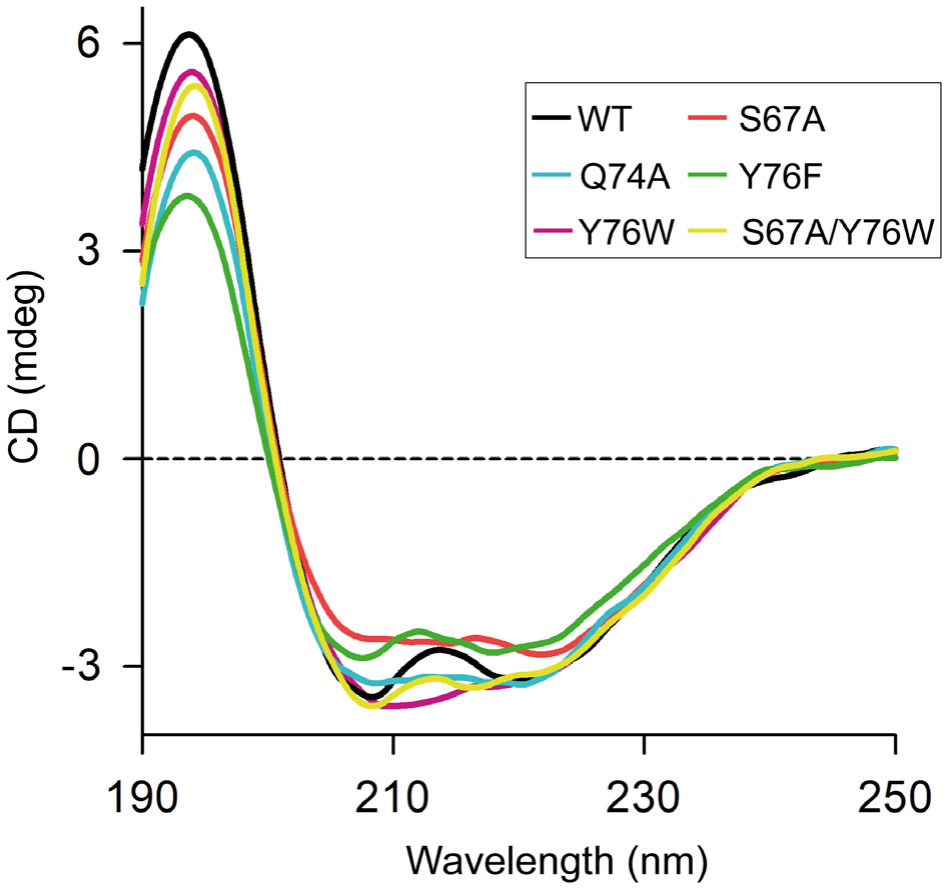
Far‐UV CD spectra of SpGrx4 WT and mutants at 4 °C.

The changes in the secondary structure were evaluated using far‐UV CD spectroscopy upon incubation of SpGrx4 WT and mutants at 4 °C for 1 h (Fig. 5 and Table S2). Among the mutants, S67A, Y76W, and S67A/Y76W showed similar α‐helical content compared with WT. By contrast, mutations slightly reduced the α‐helical content of Q74A and Y76F.

## Discussion

SpGrx4 is a class II Fe‐S transferase belonging to the Trx‐fold superfamily. The notable structural difference between the class I and II Grx members is an extension of five amino acids in the loop prior to the N‐terminal active site cysteine in the class II Grxs [9, 29]. A structural comparison of prokaryotic class I and II Grx members showed that they stabilize the cis‐proline loop differently. The glutamine residue (cis‐proline plus1) of the class I Grx3 members forms a hydrogen bond with a histidine residue on β4 (Fig. 1B and Fig. S1). A protonated histidine makes a potent proton donor compared to conventional hydrogen bond formation by neutral groups [18]. On the contrary, the corresponding glutamine residue of class II Grx4 members formed a hydrogen bond to an aromatic residue on β3 (cis‐proline plus3), directly or indirectly, depending on the temperature of the habitat. When phenylalanine appears on β3, the glutamine residue forms a hydrogen bond with a serine residue in the cis‐proline loop (Fig. 1D). While the cold‐adapted enzymes exhibit extended loop length compared with their mesophilic counterparts [30, 31], the length of the cis‐proline loop did not vary depending on the temperature of the habitat.

This study showed that the hydrogen bond network is essential for maintaining the conformational stability of the cis‐proline loop in SpGrx4. Mutants with reduced hydrogen bonds in the cis‐proline loop showed increased urea‐induced unfolding and structural flexibility of the protein (Figs 2 and 3). In particular, Q74A showed the most flexible structure among the mutants despite the highest *T*_m_ value of 56.79 °C. The structure of SpGrx4 suggested that alanine substitution (Q74A) increased hydrophobic interactions with the aromatic residues, including F21, F66, W69, Y76, and F81 (Fig. 1A). In this sense, the mutation of Q74 to Glu is considered to cause a big change in the stability as shown in the failure of Q74E expression in *E. coli*. On the other hand, Y76W with hydrogen bonds between Q74–S67–W76 showed the highest conformational stability.

As the hydrophobicity at lower and higher temperatures becomes weaker than at moderate temperatures [32], the tyrosine residue, Y76, is preferred for the cold adaptation of SpGrx4 with a heat‐labile structure (*T*_m_ = 41.52 °C) despite the rigid cis‐proline loop, due to hydrogen bond formation of Y76. By contrast, psychrophilic and mesophilic Grx4 members prefer a tryptophan residue with an indole group as shown for S67A/Y76W (*T*_m_ = 46.06 °C). In comparison, moderate thermophilic Grx4 members prefer a purely hydrophobic phenylalanine residue, as shown for Y76F (*T*_m_ = 49.68 °C). The absence of a hydrogen bond in Y76F makes the structure more vulnerable to urea‐induced unfolding and more flexible than WT. Overall, the aromatic residue on β3 (cis‐proline plus3) modulates the stability‐flexibility of the cis‐proline loop via hydrogen bonds and hydrophobic interactions.

A question arises as to why thermophilic Grx4 members do not prefer a tryptophan residue on β3, which confers thermal stability. These results suggest that a too‐rigid structure of the cis‐proline loop is not preferred for thermophilic Grx4 members, probably for the Fe‐S transferase activity, because no mutation of Y76W was found in the BLAST search. As a result, tyrosine with a phenyl hydroxyl group could be a desirable choice for Grx4 members at lower and higher temperatures to compensate for the reduced hydrophobicity via hydrogen bond formation.

**Fig. 6:**
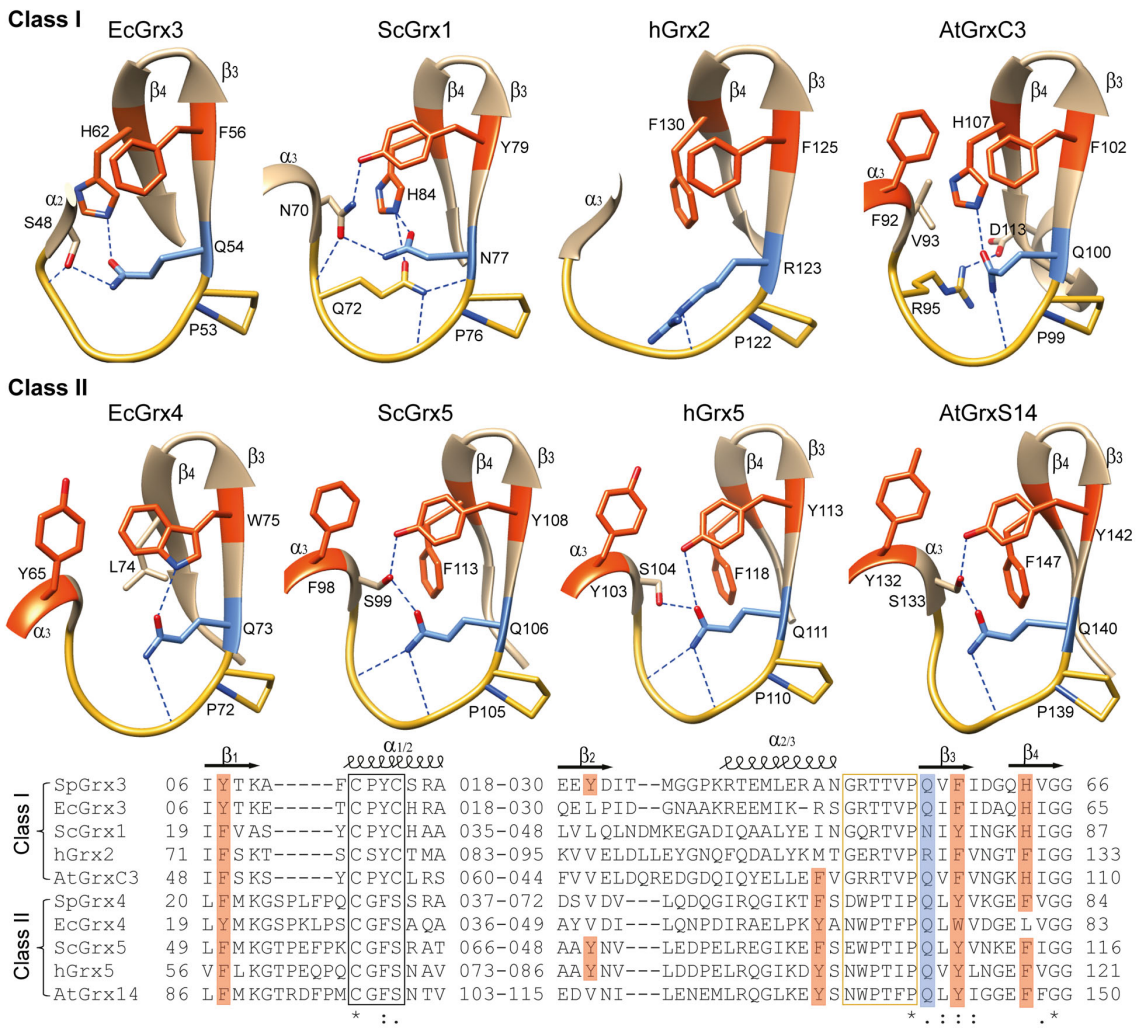
Cis‐proline loop structure of class I and class II Grx members and sequence comparison. The cis‐proline loop structures of class I Grx members (top) and class II Grx members (middle). Multiple sequence alignment of class I and II Grx members (bottom). Active site motif (black box), cis‐proline loop (yellow box), aromatic residues (orange), and cis‐proline plus1 (blue). EcGrx3 (*Escherichia coli*, PDB ID: 1FOV), ScGrx1 (*Saccharomyces cerevisiae*, PDB ID: 3C1S), hGrx2 (human, PDB ID: 2HT9), AtGrxC3 (*Arabidopsis thaliana*, AF‐Q9FVX1‐F1), ScGrx5 (*S. cerevisiae*, PDB ID: 3GX8), hGrx5 (human, PDB ID: 2WUL), and AtGrxS14 (*A. thaliana*, PDB ID: 2MMA).

The Trx‐fold of eukaryotic class I and II Grx members also showed aliphatic and aromatic clusters on either side of the central β‐sheet (Fig. 6). Eukaryotic class I Grx members exhibit different configurations for the cis‐proline loop stabilization. They also exhibited asparagine (*Saccharomyces cerevisiae*) and arginine (human) at the cis‐proline plus1 position (Fig. 6). Yeast ScGrx1 showed the hydrogen bond network patterns of both prokaryotic class I and II Grxs, in which N77 in the cis‐proline loop of ScGrx1 forms two hydrogen bonds, one with H84 on β4 and the other with Y79 on β3 via an in‐between N70 on α3 (Fig. 6) [33]. A glutamine residue (Q72) also forms a hydrogen bond with H84 in ScGrx1. On the contrary, in human Grx2, the histidine residue on β4 is replaced with a phenylalanine residue (F130), and R123 (cis‐proline plus1) forms a hydrogen bond with the cis‐proline loop mainchain (Fig. 6) [34]. *Arabidopsis thaliana* AtGrx3 showed the combination of hydrogen bond and salt bridge in the cis‐proline loop: hydrogen bonds involving the cis‐proline loop mainchain, Q100, and H107, and a salt bridge between R95 and D113 (Fig. 6). On the contrary, eukaryotic class II Grx members showed hydrogen bond networks similar to prokaryotic class II Grx members, in which the glutamine residue (cis‐proline plus1) forms a hydrogen bond with an aromatic residue on β3 (cis‐proline plus3) directly or indirectly via a serine residue (Fig. 6).

The sequences in the cis‐proline loop, except the invariant X‐Pro sequence, differ among the Trx‐fold superfamily [14]. The cis‐proline plus1 residue varies depending on the Trx‐fold superfamily, mostly small aliphatic residues (alanine) or polar residues (threonine, glutamine, and tyrosine) [14]. Although EcDsbA has alanine at the cis‐proline plus1 position [35], other Trx‐fold superfamily members have a polar residue at that position, including threonine in Trx, glutamine in Grx, and tyrosine in glutathione *S*‐transferase (GST) (Fig. S5) [14]. The T77 of EcTrx forms a hydrogen bond with the mainchain G74 [36]. The mutation of T77 in EcTrx did not affect the redox properties [15]. On the contrary, the spatial configuration of the aromatic cluster in human GST (hGST) differs from the other Trx‐fold superfamily members because GSTs have a canonical Trx‐fold like Grx3 [37]. A tyrosine residue (Y32) in the cis‐proline loop of hGST, in which Y32 comprises the aromatic cluster, forms a hydrogen bond with another tyrosine residue (Y61) on β3 (Fig. S5) [37]. Therefore, the Trx‐fold superfamily exhibits different degrees of hydrophobicity in their cis‐proline loops [14].

The subtle adjustment of aromatic residues depending on the temperatures or metabolic pathways has been found in cold‐adapted proteins [38, 39, 40, 41, 42]. Psychrophilic or thermophilic esterases prefer a tyrosine residue in the active site wall to form a hydrogen bond to a residue on the catalytic histidine loop [40, 41]. By contrast, psychrotrophic or mesophilic esterases prefer a tryptophan residue for histidine loop stabilization [39]. On the contrary, the two glucose 6‐phosphate dehydrogenase (G6PD) isozymes of psychrophilic *Sphingomonas* sp. harbor either a tyrosine or a phenylalanine residue in their substrate‐binding pockets: G6PD1 with a tyrosine residue for enzymatic activity in the Entner–Doudoroff pathway and G6PD2 with a phenylalanine residue for thermal stability in the oxidative pentose phosphate pathway [42].

In conclusion, the tyrosine residue, Y76, is preferred for the cold adaptation of SpGrx4 with a heat‐labile structure despite the rigid cis‐proline loop owing to hydrogen bond formation. An aromatic residue on β3 (cis‐proline plus3) plays a vital role in the stability‐flexibility of the cis‐proline loop for temperature adaptation of prokaryotic class II Grx4 members.

## Conflict of interest

The authors declare no conflict of interest.

## Author contributions

TH, CJ, S‐HJ, and CL conceived and designed the experiments. TH and CJ performed the experiments. TH and CL wrote the manuscript. TH, CJ, S‐HJ, and CL reviewed and edited the manuscript.

## Supporting information


**Fig. S1.** Comparison of the structure in class I and class II Grx members. The Trx‐fold delimits the hydrophobic core of Grxs into two clusters on either side of the central β‐sheet, the aliphatic cluster (green) and the aromatic cluster (orange).
**Fig. S2.** Multiple sequence alignment of class I Grx3 members. The glutamine residue on β3 (cis‐proline plus1) and the histidine residue on β4 are highly conserved in Grx3 members. The cis‐proline loop residues are shown in a yellow color box. The amino acid sequences were retrieved from NCBI or Uniprot: SpGrx3 (WP_010217562.1), PmGrx3 (WP_140678632.1), PhGrx3 (YP_338909.1), CpGrx3 (WP_011045119.1), EcGrx3 (P0AC62.2), RnGrx3 (WP_008217797.1), AfGrx3 (WP_035195182.1), CjGrx3 (QIK38813.1), PeGrx3 (TXF13737.1), and TtGrx3 (QGU33019.1).
**Fig. S3.** SDS‐polyacrylamide gel electrophoresis of SpGrx4 WT and mutants.
**Fig. S4.** Temperature melting curves for SpGrx4 WT and mutants. Derivative of fluorescence with respect to temperature (dF/dT) using SYPRO orange‐based protein thermal shift assay. Black dotted vertical lines indicate the derivative *T*m values.
**Fig. S5.** Enlarged view of the cis‐proline loop region in Trx, DsbA, and GST. Trx‐fold protein exhibit different hydrophobicity in the cis‐proline loops (yellow). Cis‐proline plus1 (blue), cis‐proline plus3 (orange), and aromatic cluster residues (cyan). hGST (PDB ID: 4GTU), EcTrx (PDB ID: 2TRX), and EcDsbA (PDB ID: 1FVK).
**Table S1.** List of primers for cloning into a TA vector and site‐directed mutagenesis.
**Table S2.** Content of α‐helix and β‐strand in SpGrx4 WT and mutants.

## Data Availability

The data that support the findings of this study are available from the corresponding author upon reasonable request.
