# Supplementary material for: Tyr76 is essential for the cold adaptation of a class II glutaredoxin 4 with a heat‐labile structure from the Arctic bacterium Sphingomonas sp
Source: FEBS Open Bio. 2023 Jan 29;13(3):500–10. doi: 10.1002/2211-5463.13560 (PMC9989929; doi:10.1002/2211-5463.13560)
Supplement: Supplementary file 1 — Fig. S1. Comparison of the structure in class I and class II Grx members. The Trx‐fold delimits the hydrophobic core of Grxs into two clusters on either side of the central β‐sheet, the aliphatic cluster (green) and the aromatic cluster (orange). Fig. S2. Multiple sequence alignment of class I Grx3 members. The glutamine residue on β3 (cis‐proline plus1) and the histidine residue on β4 are highly conserved in Grx3 members. The cis‐proline loop residues are shown in a yellow color box. The amino acid sequences were retrieved from NCBI or Uniprot: SpGrx3 (WP_010217562.1), PmGrx3 (WP_140678632.1), PhGrx3 (YP_338909.1), CpGrx3 (WP_011045119.1), EcGrx3 (P0AC62.2), RnGrx3 (WP_008217797.1), AfGrx3 (WP_035195182.1), CjGrx3 (QIK38813.1), PeGrx3 (TXF13737.1), and TtGrx3 (QGU33019.1). Fig. S3. SDS‐polyacrylamide gel electrophoresis of SpGrx4 WT and mutants. Fig. S4. Temperature melting curves for SpGrx4 WT and mutants. Derivative of fluorescence with respect to temperature (dF/dT) using SYPRO orange‐based protein thermal shift assay. Black dotted vertical lines indicate the derivative Tm values. Fig. S5. Enlarged view of the cis‐proline loop region in Trx, DsbA, and GST. Trx‐fold protein exhibit different hydrophobicity in the cis‐proline loops (yellow). Cis‐proline plus1 (blue), cis‐proline plus3 (orange), and aromatic cluster residues (cyan). hGST (PDB ID: 4GTU), EcTrx (PDB ID: 2TRX), and EcDsbA (PDB ID: 1FVK). Table S1. List of primers for cloning into a TA vector and site‐directed mutagenesis. Table S2. Content of α‐helix and β‐strand in SpGrx4 WT and mutants. [file FEB4-13-500-s001.pdf]

Supporting Information for

Tyr76 is essential for the cold adaptation of a class II glutaredoxin 4 with a heat-labile structure from the Arctic bacterium *Sphingomonas* sp.

Trang Hoang[1], ChanSu Jeong[1], Sei-Heon Jang[1], and ChangWoo Lee[1], *

[1]Department of Biomedical Science and Center for Bio-Nanomaterials, Daegu University, Gyeongsan 38453, South Korea

Table S1. List of primers for cloning and site-directed mutagenesis.

		Primer sequence
WT	Forward	5′- catatgaccgacgaatcccagac-3′ *(Nde* I site underlined*)*
	Reverse	5′- ggatcctcagttcgccttcg-3′ (*BamH* I site underlined)
S67A	Forward	5′-ggaatcaagacctttGCGgactgg-3′
	Reverse	5′-gtcggccagtcCGCaaaggt-3′
Q74A	Forward	5′-gacgatcccgGCGctgtatg-3′
	Reverse	5′-cttcacatacagCGCcgggatc-3′
Y76F	Forward	5′-cagctgTTTgtgaagggc-3′
	Reverse	5′-aattcgcccttcacAAAcagc-3′
Y76W	Forward	5′-gcagctgTGGgtgaaggg-3′
	Reverse	5′-attcgcccttcacCCAcagc-3′
S67A/Y76W	Forward	5′-ggaatcaagacctttGCGgactgg-3′
	Reverse	5′-gtcggccagtcCGCaaaggt-3′

Mutated nucleotides are shown in capital letters. The double mutant, S67A/Y76W, was generated from the primers for S67A in PCR using the nucleotide sequence of Y76W as a template.

Table S2. Content of α-helix and β-strand in SpGrx4 WT and mutants.

	α-helix (%)	β-strand (%)
WT	24.22	27.57
S67A	25.38	27.83
Q74A	22.83	26.89
Y76F	22.96	28.98
Y76W	24.97	26.79
S67A/Y76W	23.55	27.62

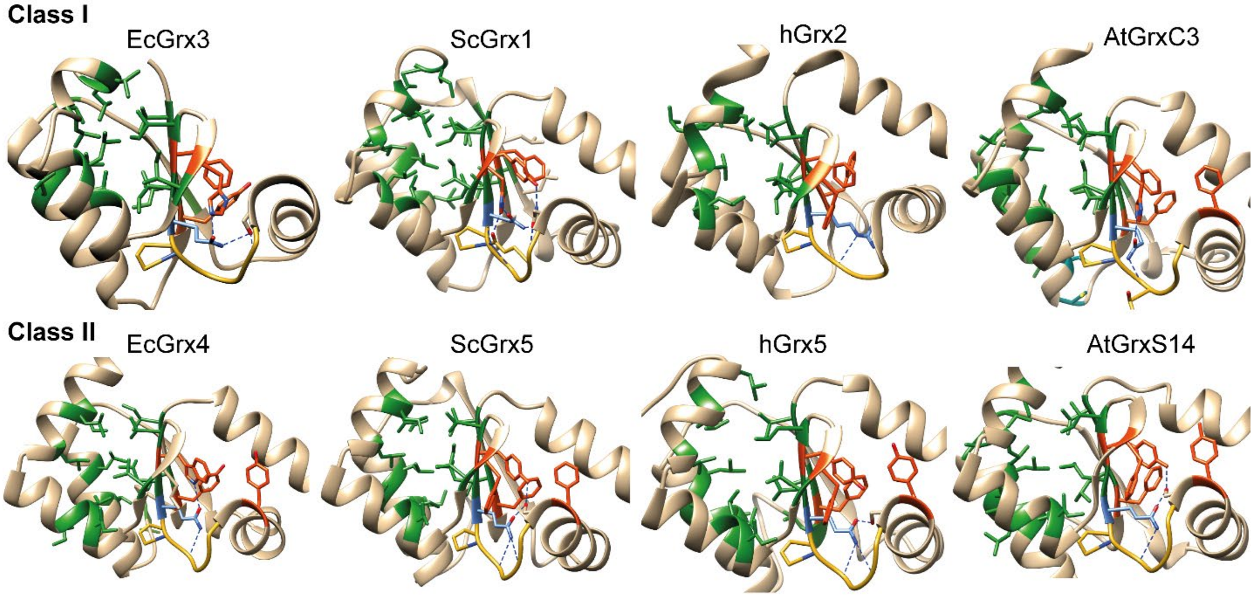

Figure S1. Comparison of the structure in class I and class II Grx members. The Trx-fold delimits the hydrophobic core of Grxs into two clusters on either side of the central β-sheet, the aliphatic cluster (green) and the aromatic cluster (orange).

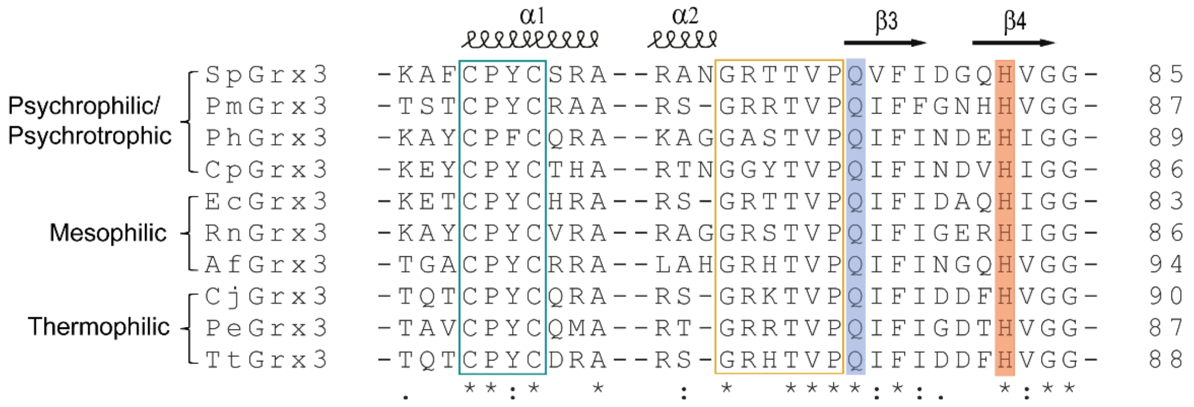

Figure S2. Multiple sequence alignment of class I Grx3 members. The glutamine residue on β3 (cis-proline plus1) and the histidine residue on β4 are highly conserved in Grx3 members. The cis-proline loop residues are shown in a yellow color box. The amino acid sequences were retrieved from NCBI or Uniprot: SpGrx3 (WP_010217562.1), PmGrx3 (WP_140678632.1), PhGrx3 (YP_338909.1), CpGrx3 (WP_011045119.1), EcGrx3 (P0AC62.2), RnGrx3 (WP_008217797.1), AfGrx3 (WP_035195182.1), CjGrx3 (QIK38813.1), PeGrx3 (TXF13737.1), and TtGrx3(QGU33019.1)

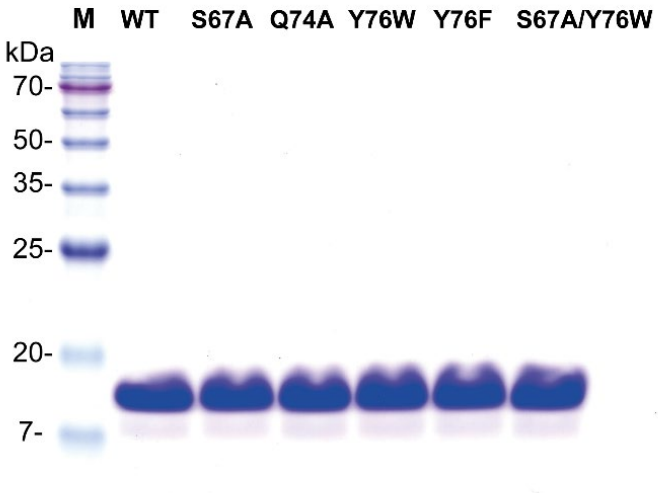

Figure S3. SDS-polyacrylamide gel electrophoresis of SpGrx4 WT and mutants.

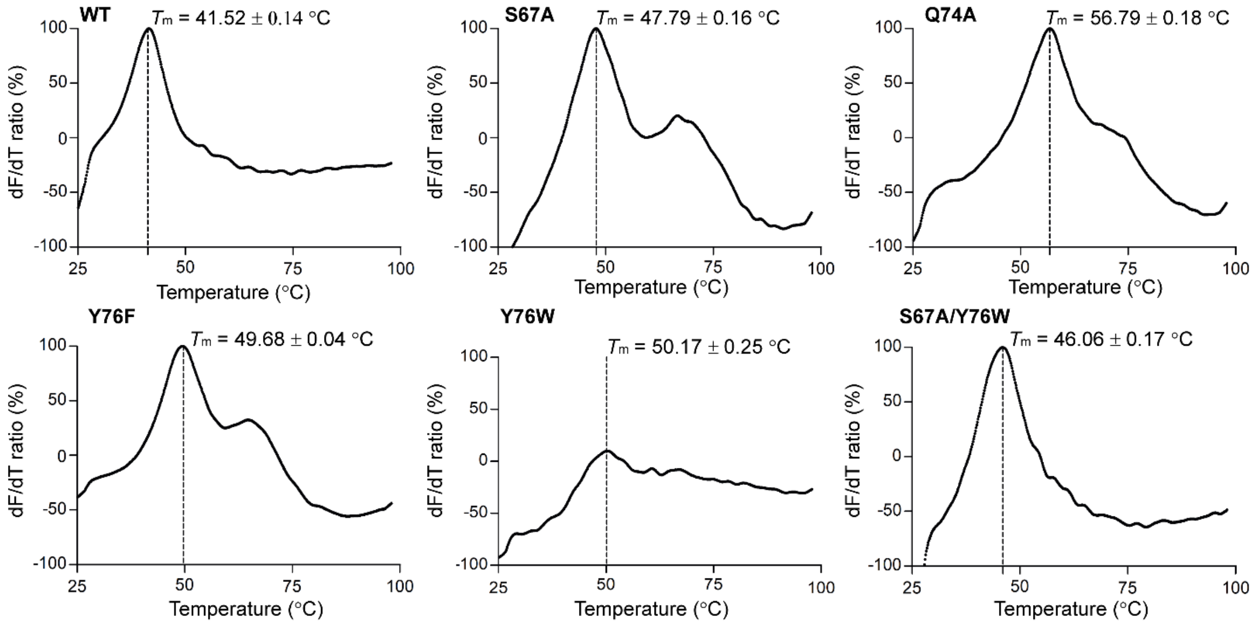

Figure S4. Temperature melting curves for SpGrx4 WT and mutants. Derivative of fluorescence with respect to temperature (dF/dT) using SYPRO orange-based protein thermal shift assay. Black dotted vertical lines indicate the derivative T_m values.

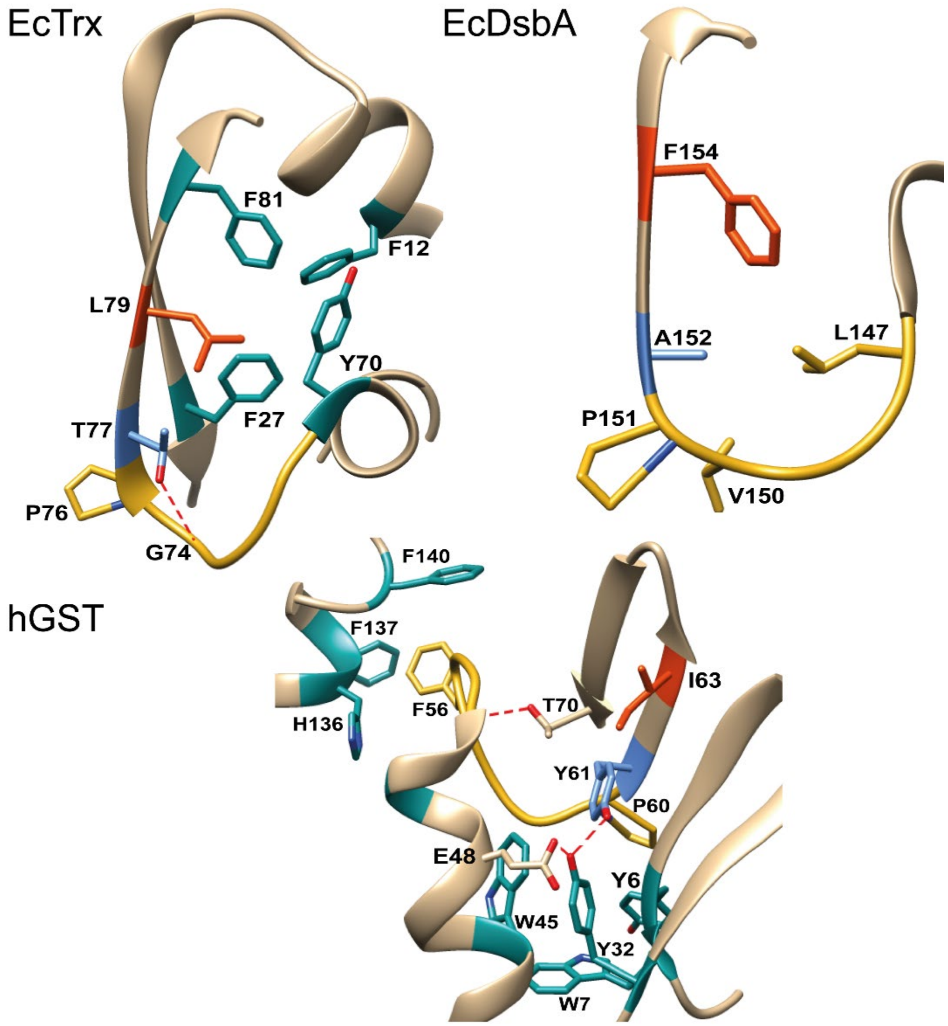

Figure S5. Enlarged view of the cis-proline loop region in Trx, DsbA, and GST. Trx-fold protein exhibit different hydrophobicity in the cis-proline loops (yellow). Cis-proline plus1 (blue), cis-proline plus3 (orange), and aromatic cluster residues (cyan). hGST (PDB ID: 4GTU), EcTrx (PDB ID: 2TRX), and EcDsbA (PDB ID: 1FVK).
